# Identification of Luminal A breast cancer by using deep learning analysis based on multi-modal images

**DOI:** 10.3389/fonc.2023.1243126

**Published:** 2023-11-17

**Authors:** Menghan Liu, Shuai Zhang, Yanan Du, Xiaodong Zhang, Dawei Wang, Wanqing Ren, Jingxiang Sun, Shiwei Yang, Guang Zhang

**Affiliations:** ^1^ Department of Health Management, The First Affiliated Hospital of Shandong First Medical University & Shandong Engineering Laboratory for Health Management, Shandong Medicine and Health Key Laboratory of Laboratory Medicine, Shandong Provincial Qianfoshan Hospital, Jinan, China; ^2^ Department of Radiology, Shandong Provincial Hospital Affiliated to Shandong First Medical University, Jinan, China; ^3^ Postgraduate Department, Shandong First Medical University (Shandong Academy of Medical Sciences), Jinan, China; ^4^ Department of Radiology, The First Affiliated Hospital of Shandong First Medical University, Jinan, China; ^5^ Department of Anorectal Surgery, The First Affiliated Hospital of Shandong First Medical University, Jinan, China

**Keywords:** molecular subtype, breast cancer, multi-modality, deep learning, mammography, MRI

## Abstract

**Purpose:**

To evaluate the diagnostic performance of a deep learning model based on multi-modal images in identifying molecular subtype of breast cancer.

**Materials and methods:**

A total of 158 breast cancer patients (170 lesions, median age, 50.8 ± 11.0 years), including 78 Luminal A subtype and 92 non-Luminal A subtype lesions, were retrospectively analyzed and divided into a training set (n = 100), test set (n = 45), and validation set (n = 25). Mammography (MG) and magnetic resonance imaging (MRI) images were used. Five single-mode models, i.e., MG, T2-weighted imaging (T2WI), diffusion weighting imaging (DWI), axial apparent dispersion coefficient (ADC), and dynamic contrast-enhanced MRI (DCE-MRI), were selected. The deep learning network ResNet50 was used as the basic feature extraction and classification network to construct the molecular subtype identification model. The receiver operating characteristic curve were used to evaluate the prediction efficiency of each model.

**Results:**

The accuracy, sensitivity and specificity of a multi-modal tool for identifying Luminal A subtype were 0.711, 0.889, and 0.593, respectively, and the area under the curve (AUC) was 0.802 (95% CI, 0.657- 0.906); the accuracy, sensitivity, and AUC were higher than those of any single-modal model, but the specificity was slightly lower than that of DCE-MRI model. The AUC value of MG, T2WI, DWI, ADC, and DCE-MRI model was 0.593 (95%CI, 0.436-0.737), 0.700 (95%CI, 0.545-0.827), 0.564 (95%CI, 0.408-0.711), 0.679 (95%CI, 0.523-0.810), and 0.553 (95%CI, 0.398-0.702), respectively.

**Conclusion:**

The combination of deep learning and multi-modal imaging is of great significance for diagnosing breast cancer subtypes and selecting personalized treatment plans for doctors.

## 1 Introduction

Breast cancer is the most common cancer in women and the second cause of death after cardiovascular diseases (1). In 2020, more than 2.2 million new breast cancer cases were diagnosed in women worldwide. In recent years, due to increased awareness of early breast cancer screening and the development of effective targeted therapy techniques, the overall mortality rate of breast cancer has decreased; however, the incidence rate continues to rise, especially in the younger population (1). Breast cancer can be classified into four molecular subtypes, i.e., Luminal A, Luminal B, human epidermal growth factor, and triple-negative breast cancer (2). Patients with different subtypes require different treatment plans and have different prognoses. The Luminal A subtype, also known as estrogen receptor-positive and progesterone receptor-positive cancer, accounts for about 40% of all breast cancers and is the most common subtype, more common in postmenopausal women with low histological grades (3). Luminal A subtype is early-stage breast cancer, less aggressive and more sensitive to endocrine therapy than Luminal B, and less sensitive to chemotherapy, with the lowest recurrence rate and the best prognosis among the four subtypes (4). Therefore, early and accurate identification of Luminal A breast cancer patients is of utmost importance.

Currently, imaging and pathological examination are the major means for diagnosing breast cancer. The most common imaging tool is mammography imaging; yet, its sensitivity tends to decrease when screening middle-aged people with higher mass density (5). Conventional magnetic resonance imaging (MRI) is also often applied; although highly sensitive, this method can potentially detect false positives (6). Pathological examinations are mainly based on examination on direct examination of breast cancer tissue collected by biopsy. Yet, the major drawbacks of this method are its invasive and limited sample collection. Thus, searching for a more accurate and less invasive breast cancer subtype screening tool is urgently needed.

In recent years, with the rapid development of artificial intelligence, deep learning has also been used to identify breast cancer molecular subtypes. Zhang et al. (7) and Sun et al. (8) used deep learning models based on breast ultrasound images and three dynamic contrast-enhanced magnetic resonance imaging (DCE-MRI) sequences to identify molecular subtypes obtaining good results. Yet, these studies were based on a single model of breast imaging.

Multi-modal imaging is a comparative analysis method that can simultaneously produce signals for more than one imaging technique, thus increasing accuracy and qualitative diagnosis of tumors through complementary and cross-validation. Recently, few studies have applied machine learning or deep learning to determine benign and malignant breast tumors based on breast multi-modal images. Li et al. (9) used a combination of digital breast tomosynthesis and mammography (MG) to improve the accuracy of the deep learning-based classification model of benign and malignant breast tumors. Hadad et al. (10) used the transfer learning method to classify benign and malignant lesions on breast MRI images with the pre-trained network based on MG images, achieving cross-modal effects. However, there is still a lack of research on deep learning in identifying breast cancer molecular subtypes based on breast multi-modal images.

The present study analyzed the value of deep learning methods in identifying molecular subtypes of breast cancer by combining X-ray and magnetic resonance multimodal images of breast cancer with AI.

## 2 Materials and methods

### 2.1 Patients

Institutional Review Board approved this study. Informed consent was waived because of the retrospective nature of the study. Anonymous clinical data were used in the analysis.

**Figure 1:**
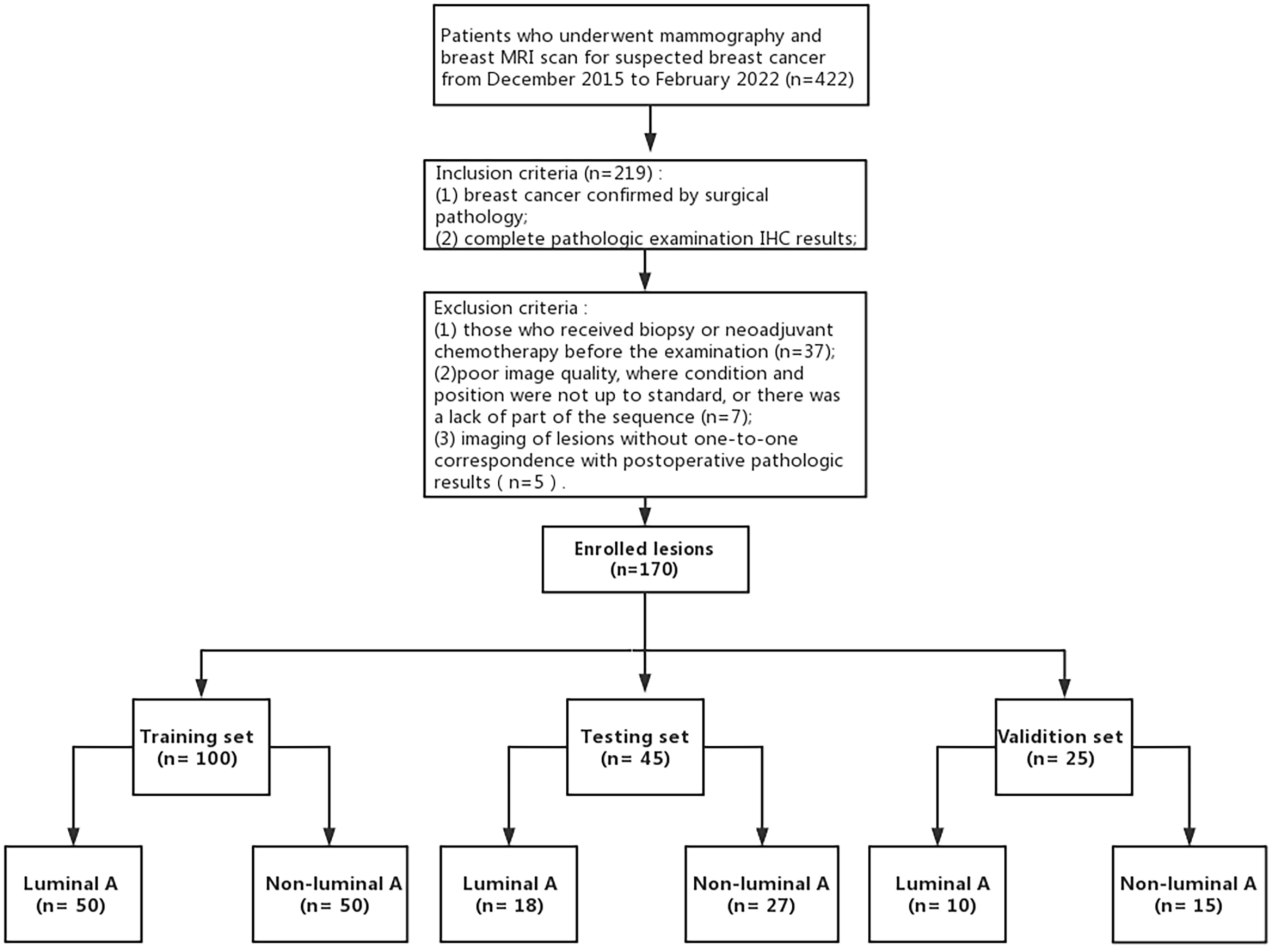
Process of enrolling patients with inclusion and exclusion criteria.

A total of 158 breast cancer patients (170 lesions) were enrolled from the First Affiliated Hospital of Shandong First Medical University. Inclusion criteria were the following (1): patients who underwent mammography and MRI scan for suspected breast cancer; (2) breast cancer confirmed by surgical pathology; (3) complete pathologic examination immunohistochemistry results. Exclusion criteria were the following: (1) those who received biopsy or neoadjuvant chemotherapy before the examination; (2) poor image quality, where condition and position were not up to standard, or there was a lack of part of the sequence; (3) imaging of lesions without one-to-one correspondence with postoperative pathologic results (**Figure 1**).

### 2.2 Mammography

MG examination was performed with digital mammography (Hologic Selenia Dimensions). During the examination, the breast was placed on the detector and flattened by the compressor. The bilateral breast’s medial and lateral-oblique and cranial-caudal images were collected. If the observation was not satisfactory, other positions, such as lateral or cleavage, were added.

### 2.3 MRI examination

Breast MRI was performed using a 3.0T MRI scanner (Magnetom Skyra) from Siemens, Germany, and a 1.5T MRI scanner (Signa Explorer) from GE, USA, with the dedicated breast coil. The patient was placed in a prone position. The following four sequences were collected by the two instruments: axial T2-weighted image (T2WI), diffusion weighting imaging (DWI), apparent diffusion coefficient (ADC) images, and DCE-MRI sequences. The parameters of T2WI sequence in 3.0T MRI scanner are as follows: repetition time (TR) = 7600 ms, echo time (TE) = 102 ms, inverse Angle = 120°, slice gap = 0.8 mm, layer thickness = 4mm, field of view (FOV) = 34 cm × 34 cm, matrix = 576 × 576. The parameters of DWI in 3.0T MRI scanner are as follows: TR = 5030 ms, TE = 56 ms, reverse Angle = 180°, slice gap = 1.1 mm, slice thickness = 5.5 mm, FOV = 13.6 cm × 5.5 cm, matrix = 224 × 224, DWI b value = 50/1000 s/mm^2^. The parameters of DCE in 3.0T are as follows: TR = 5.6 ms, TE = 1.7 ms, reverse Angle = 10°, layer thickness = 1.5 mm, FOV = 17.3cm × 8.8 cm, matrix = 512 × 512. The parameters of T2WI sequence in 1.5T MRI scanner are as follows: TR = 5269 ms, TE = 79.7 ms, inverse Angle = 160°, slice gap = 1.0 mm, layer thickness = 5 mm, FOV = 32 cm × 32 cm, matrix = 288 × 224; The parameters of DWI sequence in 1.5T MRI scanner are as follows: TR = 5722 ms, TE = 98.4 ms, slice gap = 1.0 mm, layer thickness = 5.0 mm, FOV = 32 cm × 32 cm, matrix = 128 × 128, b value of DWI = 50/800 s/mm^2^. The parameters of DCE sequence parameters in 1.5T MRI scanner are as follows: TR = 4.6 ms, TE = 2.1 ms, inverse Angle = 15°, layer thickness = 2.2 mm, FOV = 32 cm × 32 cm, matrix = 114 × 224. DCE-MRI sequence imaging was obtained after injecting 20 ml gadolinium contrast medium (Magnevist, Bayer Schering, Germany) at a rate of 4.0 ml/s. The 3.0T MRI machine had a duration of 5 minutes and 10 seconds with a total of 10 phases, and the 1.5T MRI machine had a duration of 6 minutes and 37 seconds with 10 phases.

### 2.4 Breast image analysis and region of interest (ROI) labeling

Two physicians specializing in breast imaging diagnosis with 7 years of experience who were blinded to the clinical and pathological data analyzed the breast MRI and MG images of 170 lesions, determined the location, size and boundary of tumors, evaluated the imaging characteristics of tumors, and recorded key signs. In case of disagreements, a senior doctor with 15 years of experience was invited. For breast MRI, T2WI, DWI, ADC and DCE-MRI were selected, and the sequence with the most obvious lesion enhancement contrast was selected for the DCE-MRI sequence. All lesion images were included.

**Figure 2:**
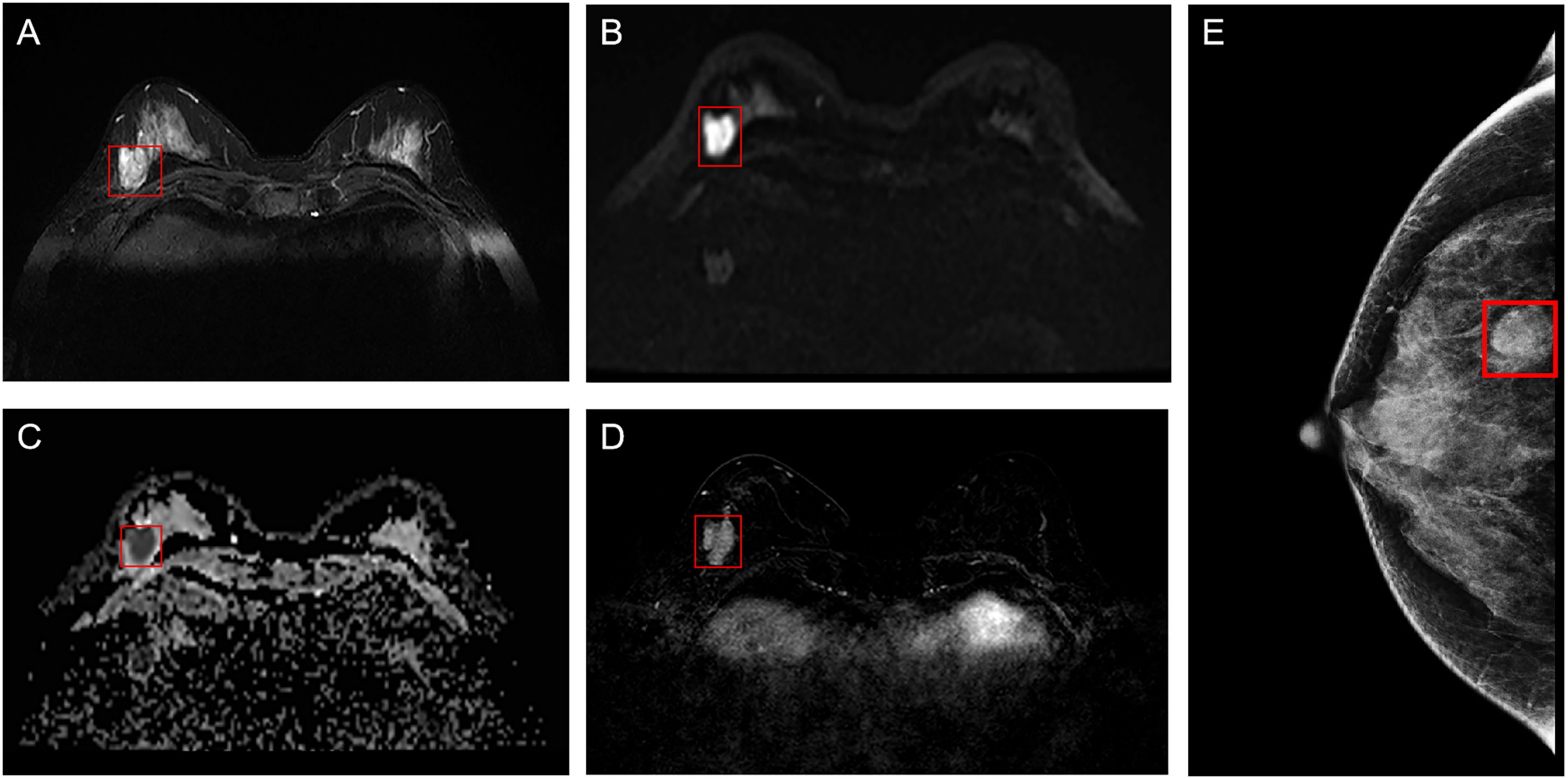
Example of ROI segmentation from raw MRI and MG images of breast cancer. **(A)** T2WI of the breast; **(B)** DWI of the breast; **(C)** ADC of the breast; **(D)** Period with the most significant enhancement in DCE-MRI of the breast; **(E)** MG of the breast. ROI, region of interest; MG, mammography; T2WI, T2-weighted imaging; DWI, diffusion weighting imaging; ADC, Apparent dispersion coefficient; DCE-MRI, dynamic contrast-enhanced magnetic resonance imaging.


ROI segmentation was performed in raw images of enrolled breast cancer lesions using the software Matlab-R2018b (Math works, Massachusetts, USA). First, the smallest square bounding box covering the tumors was determined as the input ROI for deep learning, as indicated by the radiologist analysis, as shown in **Figure 2**. Then, all the segmented ROI images were unified into a 224×224 size. Finally, the image was normalized by formula (1) so that the pixel value falls in the interval [0,1].

$$\mathrm{Norm}=\frac{\mathrm{xi}-\min\left(x\right)}{\max\left(x\right)-\min\left(x\right)}\;\;\;\;\;\left(1\right)$$

where xi represents the image pixel value, while max(x) and min(x) represent the maximum and minimum values of the image pixels, respectively.

### 2.5 Construction of deep learning model and training

**Figure 3:**
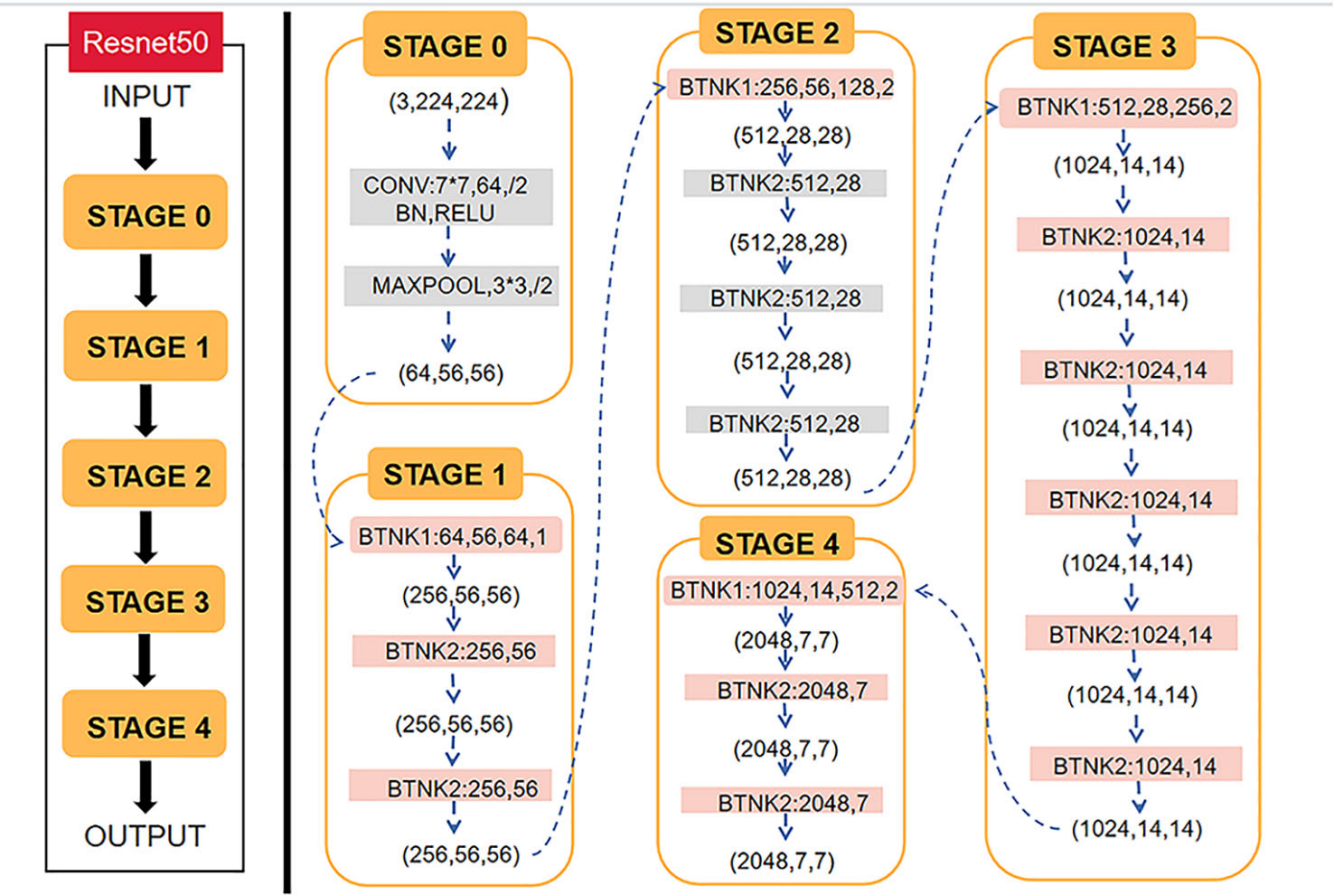
ResNet50 architecture and specific structure of each stage of ResNet50.

**Figure 4:**
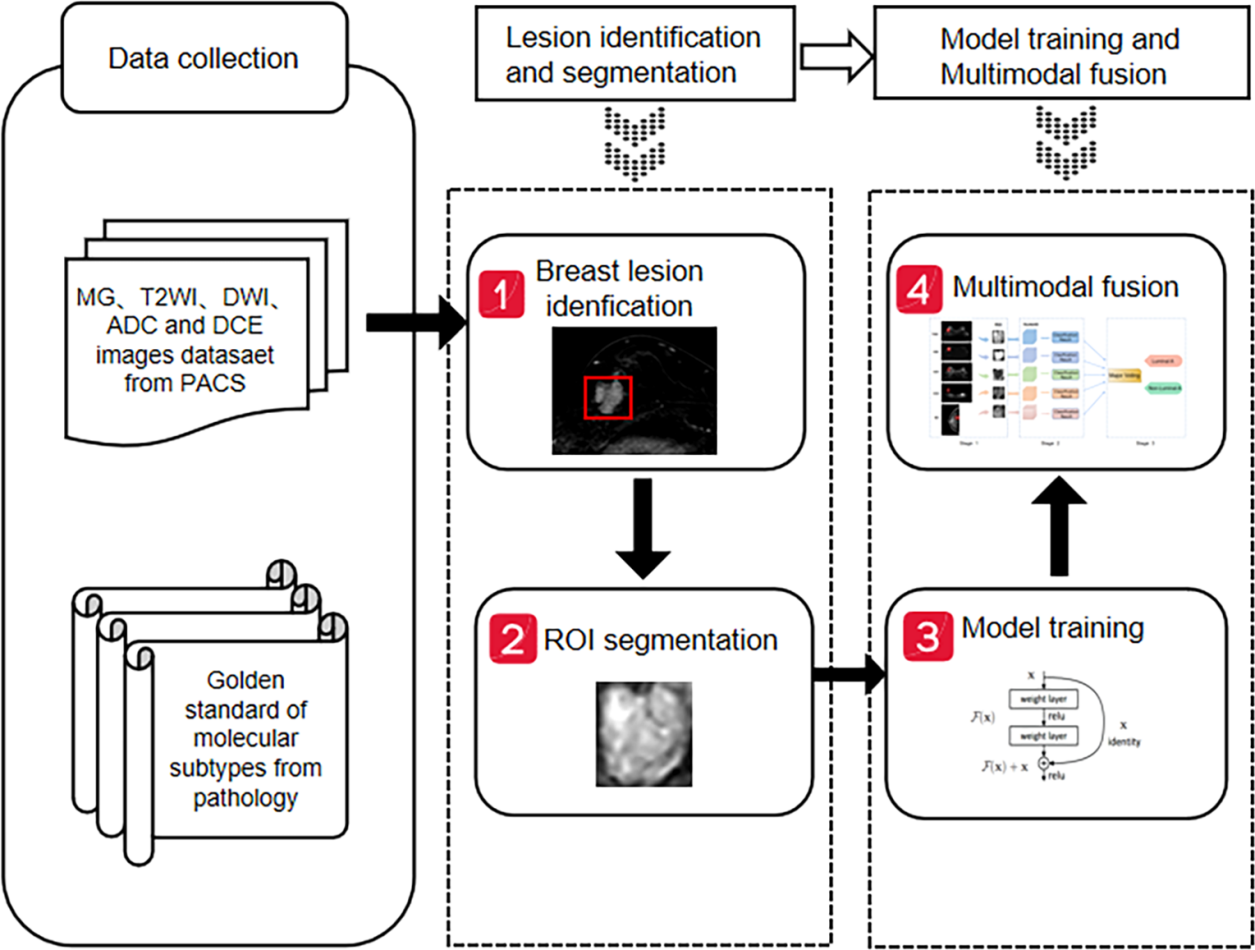
Workflow of breast cancer molecular subtypes classification.

**Figure 5:**
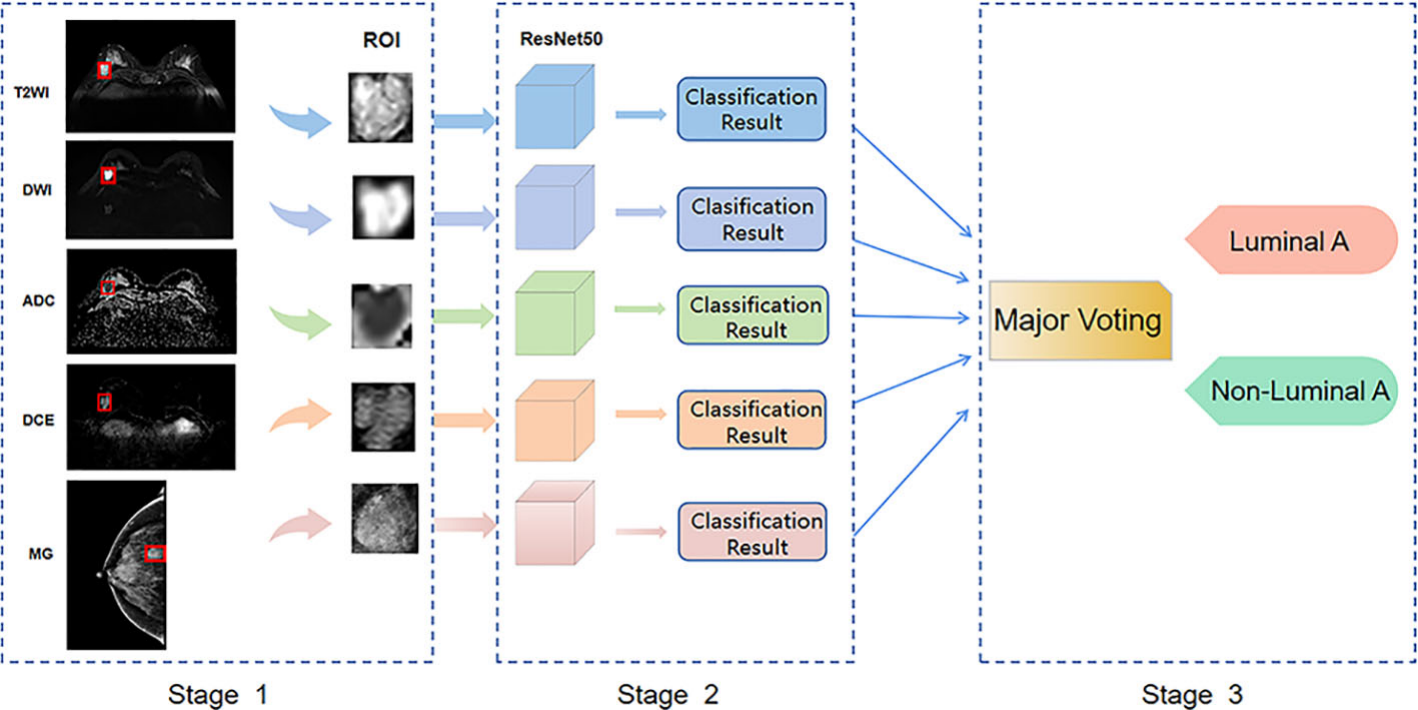
Workflow of breast cancer multi-modal fusion.

Python and the open-source deep learning library torch and math were used to construct the deep residual network (ResNet). ResNet50 architecture and specific structure are shown in **Figure 3**. The training and testing were carried out on a Windows image workstation using NVIDIA GeForce GTX 2080ti GPU for parallel computing, as follows: (1) image preprocessing was completed on Matlab_R2018b (Mathworks, Massachusetts, USA), and the annotated image input was used to extract ROI; (2) the ROI images were randomly divided into a training set (n = 100), a testing set (n = 45), and a validation set (n = 25). The training set contained 50 lesions of Luminal A and 50 lesions of non-luminal A, the testing set contained 18 lesions of Luminal A and 27 lesions of non-luminal A, and the validation set contained 10 lesions of Luminal A and 15 lesions of non-luminal A. The validation set in our study belongs to an internal validation set, in order to choose the appropriate parameters for the deep learning model. The training times epoch was set to 300 times, and the size of the training set batch_size was set to 64 frames each time. The learning rate was between 0.001 and 0.0001. (3) Data augmentation was performed on the dataset, and only the training set data was expanded, mainly by performing random geometric image transformation on the original ROI image, to expand the training samples of deep learning, which is conducive to better model generalization and prevention of overfitting. (4) Under the guidance of the theory of residual learning, the alternate connection of the residual network structure Conv Block and Identity Block not only increases the depth of the network but also solves the degradation problem of deep learning caused by the deepening of the network. Finally, the average pooling layer and the full connection layer were used to integrate the category discriminative information extracted by the previous layer. The data were input into the feature classifier Softmax for classification, and five classification models based on MG, T2WI, DWI, ADC and DCE-MRI images of the same lesion were constructed. The classifier finally outputs the predicted probability values of the image for the Luminal A subtype. When the predicted probability value of Luminal A was > 0.5, it was classified as a Luminal A subtype; when the probability value of Luminal A was< 0.5, it was judged as a non-Luminal A subtype. (5) The classification results of the five modalities were fused by the majority voting method of the idea in ensemble learning, i.e., the category with more classification results in the five modalities is output as the final classification result of the multimodal model (**Figure 4**). The multi-modal model fusion process is shown in **Figure 5**.

### 2.6 Statistical analysis

SPSS 22.0 and MedCalc 15.2.2 software were used for statistical analysis. Kolmogorov-Smirnov test was used to evaluate the normality. Quantitative data conforming to normal distribution were expressed as mean ± standard deviation, while qualitative data were expressed as frequency. An independent sample t-test was used to compare age and maximum lesion diameter differences between Luminal A and non-Luminal A lesions. χ² test was used to compare the pathological grade, lesion margin, calcification, lymph node metastasis and time-signal intensity curve (TIC) types between Luminal A and non-Luminal A patients. P< 0.05 was considered statistically significant. Confusion matrix and receiver operating characteristic (ROC) curve analysis were used to evaluate the efficiency of single - and multimodal molecular typing. DeLong test was used to evaluate the ROC curve and area under the curve (AUC) between different models, and P< 0.05 was considered statistically significant.

## 3 Results

### 3.1 General information

A total of 422 patients who underwent mammography, breast MRI scan and enhancement examination for suspected breast cancer between December 2015 and February 2022 were included in the study. Among these, the breast cancer patients confirmed by surgical pathology and who completed pathologic examination IHC results (n = 219) were included in the study. Moreover, 49 patients were excluded because of the following reasons: receiving biopsy or neoadjuvant chemotherapy before the examination (n = 37), poor image quality, condition, and position were not up to standard, lack of part of the sequence (n = 7), imaging of lesions without one-to-one correspondence with postoperative pathologic results (n = 5). Finally, a total of 158 breast cancer patients (170 lesions, median age, 50.8 ± 11.0 years), among which 12 females had 2 lesions, were included in the final analysis. Pathological types included 146 lesions of invasive breast cancer, 12 of ductal carcinoma in situ, 5 of papillary carcinoma, 4 of non-invasive lobular tumor, and 3 of mucoepidermoid carcinoma. After the immunohistochemistry examination, patients were divided into 78 Luminal A subtype cases and 92 non-Luminal A subtype cases (including the 60 Luminal B lesions, 11 HER-2 lesions, and 21 triple-negative lesions). Compared with non-Luminal A subtype, Luminal A subtype lesions had a smaller tumor size (2.0 ± 0.8 cm versus 2.5 ± 1.4 cm; p = 0.007), a higher prevalence of old age (53.0 ± 11.7 years versus 48.9 ± 10.0 years; p = 0.015), a lower prevalence of axillary lymph node metastasis (ALMN) (21.8% versus 42.4%; p = 0.001), a lower pathological grade (I 39.7% versus 12.0%, II 56.4% versus 63.0%, III 3.8% versus 25.0%; p< 0.001).There were statistically significant differences in the age of breast cancer onset, the maximum diameter of the lesion, pathological grade and lymph node metastasis between the two groups (all P< 0.05), while the margin of the lesion, calcification, and TIC type were similar (all P > 0.05) (**Table 1**).

**Table 1:** Population characteristics.

Characteristics	Total n = 170	Luminal A n = 78	Non-Luminal A n = 92	P
Age, years	50.8 ± 11.0	53.0 ± 11.7	48.9 ± 10.0	0.015
Tumor size, cm	2.2 ± 1.2	2.0 ± 0.8	2.5 ± 1.4	0.007
Calcification	95 (55.9)	42 (53.8)	53 (57.6)	0.622
ALNM	56 (32.9)	17 (21.8)	39 (42.4)	0.004
Tumor margin				0.929
Regular	66 (38.8)	30 (38.5)	36 (39.1)	
Irregular	104 (61.2)	48 (61.5)	56 (60.9)	
TIC curve type				0.939
II	91 (53.5)	42 (53.8)	49 (53.3)	
III	79 (46.5)	36 (46.2)	43 (46.7)	
Pathological grade				<0.001
I	42 (24.7)	31 (39.7)	11 (12.0)	
II	102 (60.0)	44 (56.4)	58 (63.0)	
III	26 (15.3)	3 (3.8)	23 (25.0)	

Continuous variables are described as mean ± standard deviation (SD), and categorical variables are presented as numbers (%). ALMN, axillary lymph node metastasis; TIC, time-intensity curve.

### 3.2 Diagnostic efficacy of single-mode and multi-mode models in identifying molecular subtypes of breast cancer

Five models, i.e., MG, T2WI, DWI, ADC, and DCE-MRI, were applied for each patient. The accuracy, sensitivity, specificity, and AUC value of the model based on MG images were 0.533, 0.667, 0.444, and 0.593 (95%CI, 0.436-0.737), respectively; the accuracy, sensitivity, specificity, and AUC value of T2WI models were 0.667, 0.833, 0.556, and 0.700 (95%CI, 0.545-0.827), respectively; the accuracy, sensitivity, specificity, and AUC value of DWI image prediction were 0.060, 0.722, 0.519, and 0.564 (95%CI, 0.408-0.711), respectively; the accuracy, sensitivity, specificity and AUC value of ADC image prediction were 0.622, 0.722, 0.556, and 0.679 (95% CI, 0.523-0.810), respectively; the accuracy, sensitivity, specificity, and AUC value of DCE-MRI images were 0.667, 0.556, 0.741, and 0.553 (95%CI, 0.398-0.702), respectively; the accuracy, sensitivity, specificity, and AUC value of the multimodal fusion model were 0.711, 0.889, 0.593, and 0.802 (95%CI, 0.657-0.906), respectively.

**Figure 6:**
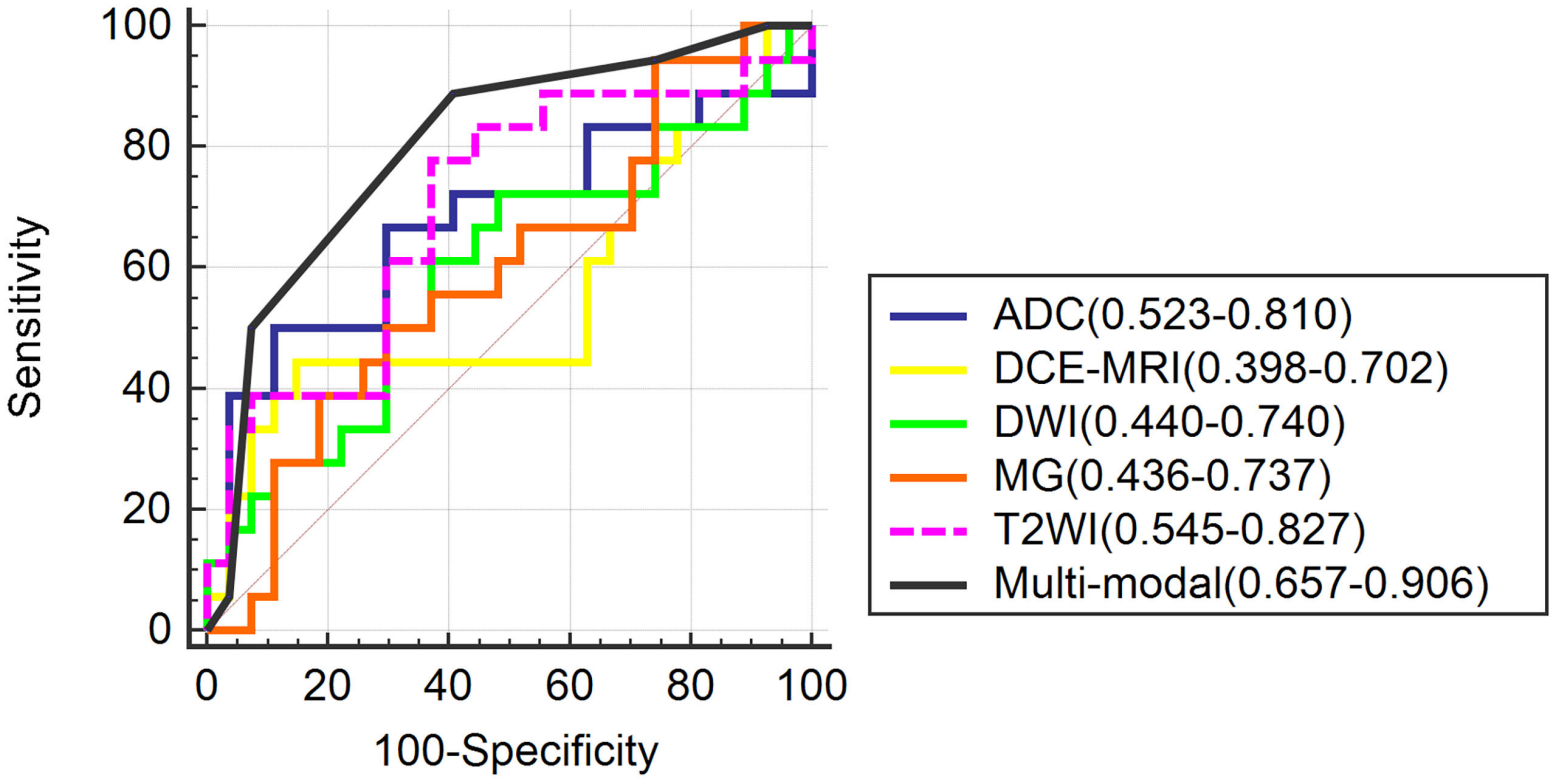
The receiver operating characteristic curves of the single models and multi-modal.

Among the five single-mode models, the accuracy, sensitivity, specificity and AUC values of T2WI models were optimal, with an accuracy of 0.667 and an AUC of 0.700 (95%CI, 0.545-0.827). Yet, the multi-modal model had the best diagnostic performance in discriminating Luminal A and non-Luminal A breast cancer, with higher accuracy and sensitivity than any single-modal model but slightly lower specificity than the DCE-MRI model, as shown in **Table 2**; the AUC value obtained by the five single- modalities (MG, T2WI, DWI, ADC, and DCE-MRI) and multi-mode model was (0.593, 0.700, 0.564, 0.679, and 0.553) and 0.802, respectively, as shown in **Table 2**. The results showed that the AUC value of the multi-modal model was higher than that of any of the five single modalities, and the differences between the AUC values of a multi-modal model with MG, DWI, and DCE-MRI were statistically significant (P< 0.05). However, the differences between the AUC values of the multimodal model, the T2WI model, and the ADC model were not obvious (P > 0.05), as shown in **Figure 6**.

**Table 2:** Diagnostic performance of the single models and multimodal.

Modality	ACC	SEN	SPE	AUC (95%CI)	P Value*
MG	0.533	0.667	0.444	0.593 (0.436-0.737)	<0.05
T2WI	0.667	0.833	0.556	0.700 (0.545-0.827)	0.2882
DWI	0.060	0.722	0.519	0.597 (0.440-0.740)	<0.05
ADC	0.622	0.722	0.556	0.679 (0.523-0.810)	0.2778
DCE-MRI	0.667	0.556	0.741	0.553 (0.398-0.702)	<0.05
Multi-modal	0.711	0.889	0.593	0.802 (0.657-0.906)	–

MG, mammography; T2WI, T2-weighted imaging; DWI, diffusion weighting imaging; ADC, apparent dispersion coefficient; DCE-MRI, dynamic contrast-enhanced magnetic resonance imaging; ACC, accuracy; SEN, sensitivity; SPE, specificity; AUC, area under the receiver operating characteristic curve; CI, confidence interval.

*The P-value is the result meaning of comparing the AUC of each single modal and multi-modal according to the Delong’s test.

## 4 Discussion

In the present study, we found significant differences in the treatment and prognosis of patients with different molecular subtypes of breast cancer. Differentiating Luminal A breast cancer from non-Luminal A molecular subtype is very important to guide clinical treatment and improve prognosis. Although several diagnostic methods have been developed, the accuracy and sensitivity of those tools for differentiating breast cancer subtypes need to be further improved. This study used a deep learning model based on multimodal imaging (mammography plus MRI) to distinguish Luminal A from non-Luminal A molecular subtypes, and good diagnostic efficacy was achieved, which was superior to MG and MRI modality alone. Therefore, the deep learning method has a certain value in the differential diagnosis of molecular subtypes of breast cancer, and multimodal image information can complement each other, providing a new idea for predicting molecular subtypes of breast cancer.

The general clinical data of breast cancer have a certain role in the differentiation of molecular subtypes of breast cancer. In this study, patients with Luminal A breast cancer showed smaller maximum diameter, lower pathological grade, and fewer axillary lymph-node metastasis than non-A breast cancer, suggesting a less aggressive type of tumor, which is consistent with the results of Szep et al. (11). However, no differences in imaging features such as tumor margin, calcification and TIC type were found, which may be related to the non-A type, including the other three subtypes and the unbalanced distribution of molecular subtypes in the enrolled patients. MG and MRI features of breast cancer with different molecular subtypes are different, which is helpful for the preliminary prediction and analysis of molecular subtypes. Other studies have found that Luminal A subtype patients’ tumor margins are more irregular than those of triple-negative breast cancers, with MG presenting stellar-shaped edges (12) and MRI presenting burr edges and unclear boundaries. Also, intralesional dark internal septation and no edema around the lesion were observed in patients with the Luminal A subtype, while type II TIC was more common (13, 14). However, these methods rely on limited human-extracted clinical and imaging features. More studies on deeper imaging features invisible to the naked eye are necessary.

Computer-aided diagnosis based on artificial intelligence has become a hot field in medical imaging research. At present, the reports on the identification of molecular subtypes of breast cancer based on artificial intelligence have mainly focused on radiomics. For example, machine learning technology has been used to extract radiomics features from MG, ultrasound, and DCE-MRI to establish a model that can non-invasively and quantitatively predict molecular subtypes, but the accuracy must be improved (15–18). Deep learning is a feature learning method in machine learning, which simulates the mechanism of the human brain neural network and converts input data into multiple abstract layers in the deep neural network that can automatically learn the required abstract deep features (19). It does not require the feature extraction steps of traditional machine learning, thus reducing the dependence on the artificial selection of key features. It can also directly achieve the end-to-end effect and may improve the accurate discrimination ability of breast cancer subtypes.

So far, a few studies have reported on molecular typing based on deep learning methods using MRI data sets (20–24). For example, a previous study (25) found that MRI-enhanced features and textures contribute to identifying molecular types of breast cancer. Ha et al. (21) demonstrated that combining deep learning-enhanced MRI images and immunohistochemical indicators is useful for identifying breast cancer subtypes, as it provides a reliable basis for the treatment, management, follow-up and prognosis of breast cancer patients. Zhang et al. (22) designed a hierarchical learning structure based on convolutional neural network, which achieved a sensitivity of 0.750 and a positive prediction rate of 0.773 in tumor segmentation. This model can be used for molecular typing of breast cancer to distinguish Luminal A breast cancer from the other three subtypes at the same time. Also, the study compared the diagnostic performance of the model with the reading results of four radiologists and concluded that the performance of the model was equal or even better than that of the radiologists. Moreover, Zhu and colleagues (23) used the transfer learning method in their study to identify Luminal A type and non-Luminal A with the pre-trained VGGNet on ImageNet, and the AUC was 0.64. Zhang et al. (24) used the DCE-MRI sequence, and based on traditional convolutional neural network and convolutional long short-term memory network, the accuracy of the deep learning model was significantly improved by the transfer learning method. Yet, the above studies only used MRI images as the research object, while the deep learning model of multi-modal images is still lacking.

In the present study, a deep learning network based on ResNet50 was used to combine the multi-modal images of MG and MRI. The accuracy, sensitivity, and AUC value of the multi-modal model were higher than those of any single-modal model, but the specificity was slightly lower than that of the DCE-MRI model. Sensitivity refers to the accuracy of the true-positive prediction of the Luminal A type, while specificity refers to the accuracy of the true-negative prediction of the non-Luminal A type. The purpose of this study was to identify Luminal A breast cancer, and non-Luminal A breast cancer includes three molecular subtypes. Therefore, as an evaluation index of diagnostic ability, sensitivity was of great significance in this study, while the evaluation of specificity was limited. Meanwhile, we found that in the five single models, the AUC of the T2WI model was relatively high, almost close to the result of the multi-modal model, which indicates that the T2WI model is relatively effective in identifying Luminal A subtype breast cancer among the deep learning models based on single-mode images. The reasons may be as follows: first, the T2WI sequence is sensitive to identifying Luminal A subtype breast cancer. T2WI images can clearly show the necrosis and low signal separation in breast cancer lesions and determine whether there is peritumoral edema (14), which is consistent with the results of Gao et al. (26). Second, the Resnet50 neural network used in this study may be more suitable for T2WI images and can extract more in-depth features. In our previous study (27), T2WI combined with the Resnet50 network model also showed superior performance in predicting breast cancer lymph node metastasis. While T2WI model showed good perfomance in the single models, the accuracy, sensitivity, and specificity of the multi-modal model were higher than that of the T2WI model. In comprehensive evaluation, the multi-modal model was still a better performing model. On the other hand, and our study also found that the specificity of the DCE-MRI model showed comparative advantages in the five single models, which also suggests the potential of the DCE-MRI model in differentiating three types of non-Luminal A breast cancer, and will be the focus of our further research. Compared with the practical application of radiologists, the diagnostic efficacy of DWI, ADC and DCE-MRI models was relatively low. This may be related to the low spatial resolution and signal-to-noise ratio of DWI and ADC images and the fact that the DCE-MRI model only selects one phase image with enhancement. The Resnet50 network may have difficulty extracting enough information from these three modalities.

Convolutional neural network (CNN) is currently the most commonly used network for deep learning in image analysis applications. In 1993, CNN was firstly introduced for medical image analysis (28). Early CNNs were relatively shallow, but demonstrated the feasibility of their ability to analyze medical images. In 2012, Hinton et al. (29) designed a CNN with five convolutional layers (also known as the “AlexNet”) that won the ImageNet Large-scale Visual Recognition Challenge with a far higher accuracy rate. Due to the breakthrough performance of AlexNet, a wide upsurge of deep learning has been set off in the academic community. VGG (Visual Geometry Group) network is a pre-trained CNN model proposed by Simonyan of Oxford University in 2014 (30). VGG pre-trained on the ImageNet dataset, which contains 1.3 million images across 1,000 categories, 100,000 for training and 50,000 for validation. The structure of VGGNet is very simple. The model consists of highly connected convolutional and fully-connected layers which enables better feature extraction and, the use of Maxpooling (in the place of average pooling) for downsampling prior classification using SoftMax activation function. But the disadvantage is that it consumes more computing resources and uses more parameters, which leads to more memory usage.

In this study, we selected ResNet50 as the basic network to conduct the deep learning model. ResNet50 is a 50-layer deep convolutional neural network. Generally, deep networks can extract more abstract information from low-level feature maps, which enables them to perform better than shallow networks (31). The residue strategy of ResNet provides a skip connection to solve the degradation problem, making it possible to train a very deep network (31). Meanwhile, ResNet has smaller parameters, faster speed and higher accuracy, which provides more feasibility for advanced feature extraction and classification. To make full use of the multi-modal image features, ResNet50 was used as the basic network for feature extraction in our method. At present, it has been used in many breast cancer image classifications. Al-Tam (32) et al. utilized the ResNet50 to identify benign and malignant breast issue. In our latest work (27), Resnet50 network model also got good result in predicting breast cancer lymph node metastasis. Therefore, we chose 50-layer ResNet for this deep learning multi-modal imaging.

In this study, we adopted the idea of ensemble learning and performed multi-modal fusion on the diagnostic results of five single modalities, i.e., MG, T2WI, DWI, ADC and DCE-MRI, which were constructed using a deep learning network. Ensemble learning requires training multiple individual learners and combining multiple individual learners to form a powerful learner through a certain combination strategy. Its advantage is that the classification results of different models are independent and do not affect each other, and the judgment errors of a single model do not cause further accumulation of errors. The majority voting method in the ensemble learning strategy adopted in this study was based on the results of five single-mode classification models and adopted the principle of the obedience of the minority to the majority to determine the category label predicted by the model. In this study, the ensemble learning method was used to combine the five modalities, which made full use of the image information of each sequence and complemented and verified the information of different modalities. It improved the accuracy of identifying breast cancer molecular subtypes and was more in line with the clinical application of radiologists.

The present study has some limitations: (1) the classification proposed in this paper only focused on Luminal A and non-Luminal A breast cancer; thus, it cannot accurately distinguish the four subtypes, which is also the common limitation of most of the studies based on deep learning in breast cancer molecular typing mentioned above; (2) This was a retrospective analysis with a relatively small sample size. For our next work, we plan to use a multi-center external validation dataset and prospective validation to further confirm these findings.

These data suggest that the deep learning method has a certain value in the differential diagnosis of molecular subtypes of breast cancer, and multimodal image information can complement each other, providing a new idea for predicting molecular subtypes of breast cancer.

## Data availability statement

The raw data supporting the conclusions of this article will be made available by the authors, without undue reservation.

## Ethics statement

The studies involving humans were approved by the ethics committee and institutional review of Shandong Qianfoshan Hospital. The studies were conducted in accordance with the local legislation and institutional requirements. The ethics committee/institutional review board waived the requirement of written informed consent for participation from the participants or the participants’ legal guardians/next of kin because of the retrospective nature of the study.

## Author contributions

ML: Conceptualization, Writing-Original Draft, Writing-Review & Editing; SZ: Conceptualization, Writing-Original Draft; YD: Investigation, Data Curation, Project administration; XZ: Formal analysis, Funding acquisition; DW: Data Curation, Project administration; WR: Resources, Investigation; JS: Validation; SY: Methodology, Software; GZ: Resources, Visualization, Supervision, Funding acquisition. All authors contributed to the article and approved the submitted version.
